# Screening the Pandemic Response Box identifies novel ligands of the *Staphylococcus aureus* protein arginine kinase, McsB

**DOI:** 10.1007/s11033-025-10545-9

**Published:** 2025-05-06

**Authors:** Ryan Chetty, Alexandré Delport, Sandile Mthembu, Clinton G. L. Veale, Raymond Hewer

**Affiliations:** 1 Discipline of Biochemistry, School of Life Sciences, University of KwaZulu-Natal, Pietermaritzburg, 3201 South Africa; 2 Department of Chemistry, University of Cape Town, Rondebosch, Cape Town, 7701 South Africa

**Keywords:** McsB, ClpCP, *Staphylococcus aureus*, TSA and CETSA

## Abstract

**Background:**

The protein arginine kinase, McsB, plays a pivotal role in the stress-response mechanism of gram-positive bacteria and represents a potential target to combat gram-positive pathogens. There are currently no recorded ligands or inhibitors reported for bacterial McsB.

**Methods and results:**

We sought to identify novel ligands for the *Staphylococcus aureus* McsB by screening the Pandemic Response Box using thermal shift and cellular thermal shift assays. Six compounds were identified as McsB ligands, inducing positive shifts in the melting and aggregating temperature of the protein. Compounds MMV1593539 and MMV1782355 imparted the greatest stability to McsB across both assays. While none of the six McsB-targeting ligands yielded anti-bacterial effect against *S. aureus* under standard or heat stress conditions, MMV1634391, MMV1633968 and MMV1782213 effectively potentiated the activity of ciprofloxacin. Molecular docking and dynamic studies predict the ATP pocket of McsB as the likely binding site for MMV1593539 and MMV1782355.

**Conclusions:**

Compounds MMV1593539 and MMV1782355 stabilised McsB in two thermal stability assays while returning the most favourable docking scores and retaining protein-ligand stability in molecular dynamics. These ligands signify promising candidates for future drug discovery efforts aimed at inhibiting or exploiting the protein arginine kinase, McsB.

**Supplementary Information:**

The online version contains supplementary material available at 10.1007/s11033-025-10545-9.

## Introduction

McsB is a protein arginine kinase that serves as an integral component of the stress response system of gram-positive bacteria along with the transcriptional repressor CtsR and the ClpC: ClpP (ClpCP) ATPdependent protease complex [1–4]. Through its unique arginine phosphorylation activity, McsB controls transcriptional and post-transcriptional processes central to stress signalling and protein quality control mechanisms. One major substrate for McsB is CtsR and, in the first instance, McsB phosphorylates arginine residues in the DNA binding domain of CtsR, promoting the dissociation of this transcriptional repressor from DNA. This allows for the expression of genes regulated by CtsR including those that encode for heat shock proteins and protein quality control factors such as McsB, ClpCP and CtsR itself [1, 2]. Accordingly, McsB-mediated arginine phosphorylation of CtsR regulates expression of the core components of the bacterial stress response system. In the second instance, McsB serves as a specialized degradation labeller through the selective phosphorylation of unfolded or misfolded proteins. The octameric form of McsB, enriched along with other higher order oligomers under stress conditions, assembles into a self-compartmentalized kinase inaccessible to natively-folded proteins [3]. Unfolded polypeptides that enter the McsB kinase chamber through lateral entry gates are phosphorylated on arginine’s and the resulting pArg-labelled polypeptides are recognised and subsequently degraded by the ClpCP complex. Through a role conceptually similar to that of E3 ligase in the eukaryotic ubiquitin proteasomal system, McsB directs bacterial protein quality control through this pArg-dependent degradation system.

While protein phosphorylation is a ubiquitous post-translational modification found across all domains of life, protein arginine phosphorylation appears to be less prevalent. Apart from a small number of pArg proteins reported in eukaryotes [5, 6], the arginine phosphoproteome is currently limited to the ~ 300 substrates found in gram-positive bacterial species [7, 8]. As a consequence of its critical and unique function, McsB has been proposed as a potential drug target for virulent gram-positive bacterial strains [3]. Inhibition of McsB function- whether through active site modulation or, as was previously proposed by Hajdusits and coworkers following their delineation of the single phosphorylation event that stabilizes the relevant form [3], by disrupting oligomeric conversion - would have significant bearing on bacterial survival. Alternatively, McsB degradation power could be harnessed, *via* PROTAC or BacPROTAC equivalents, to instigate cell death through targeted proteolysis of essential proteins. For either approach, small molecule ligands would be the requisite starting point for the development of McsB-targeting antibiotics. To date, however, no small molecule ligands or inhibitors of McsB have been described in literature.

The Pandemic Response Box was designed through collaboration between Medicines for Malaria Venture (MMV) and Drugs for Neglected Diseases initiative (DNDi) to increase the rate of discovery of novel treatments for life-threatening pandemic diseases [9, 10]. The box, available freely through the MMV or DNDi, consists of a collection of 400 structurally-diverse compounds comprising 201 anti-bacterial, 153 anti-viral and 46 anti-fungal compounds, all of which are either in the clinic or currently under investigation [9, 10]. Similar to the MMV open-access initiatives that preceded it– specifically the Malaria Box and the Pathogen Box- this collection of drug-like compounds has provided a good foundation for identifying novel small molecules with activity against a range of infectious agents, including the ESKAPE pathogens [9, 11].

The distinct ability of McsB to phosphorylate arginine residues, combined with its significant role in stress response, has evoked interest in the protein as a potential drug target to combat grampositive bacteria, specifically the ESKAPE pathogens *Enterococcus faecium* and *Staphylococcus aureus* [4, 12, 13]. The absence of specific ligands or inhibitors of this disease-relevant protein, and limited knowledge of probable binding sites, has restricted efforts to probe McsB as a druggable anti-microbial target. To this end, our study identified six novel McsB ligands from the Pandemic Response Box using thermal stability measurements. We further propose the putative binding site of two of the identified ligands through molecular modelling and molecular dynamics (MD). These ligands represent important scaffolds for stimulating further drug development efforts aimed at targeting the protein arginine kinase, McsB.

## Materials and methods

### Plasmid synthesis and transformation of *Staphylococcus aureus* McsB

The gene sequence coding for full length *S. aureus* McsB (UniProt Accession Number: Q2G0P6-MCSB_STAA8; GeneID: 3920416) was synthesised and cloned into a pET-28a(+) plasmid by GenScript (New Jersey, USA). The lyophilized plasmid DNA was resuspended in Milli-Q water, and 2 ng was used to transform chemically competent *Escherichia coli* (*E.coli*) BL21(DE3) cells. Transformed cells were plated on 2xYT agar plates (16 g/L tryptone, 10 g/L yeast extract, 5 g/L NaCl, 15 g/L agar) supplemented with 50 µg/ml kanamycin and incubated overnight at 37 °C.

### Recombinant McsB expression and purification

*E. coli* cells harbouring the McsB plasmid were cultured in 2xYT broth (16 g/L tryptone, 10 g/L yeast extract, 5 g/L NaCl, 50 µg/ml kanamycin) at 37 °C until reaching mid-log phase (OD_600_ = 0.5). Protein expression was induced with 0.5 mM IPTG, followed by incubation at 18 °C for 18 h with shaking. Cells were harvested by centrifugation (5000 *x g*, 4 °C, 10 min), and the pellet resuspended in lysis buffer (50 mM sodium phosphate, 300 mM NaCl, pH 7.4) supplemented with 1 mg/ml lysozyme and 1 mM PMSF. Cell lysis was performed by sonication, and the soluble fraction was collected by centrifugation (10 000 x g, 4 °C, 1 h). For purification, the clarified lysate was incubated with Ni-NTA agarose beads (Thermo Fisher Scientific, USA) and bound proteins were eluted with 300 mM imidazole in equilibration buffer (50 mM sodium phosphate, 300 mM NaCl, pH 7.4). Eluted fractions were analysed using SDS-PAGE and western blot under non-reducing conditions. For immunodetection, the blot was probed with anti 6x-His Tag mouse monoclonal primary antibody (1: 5000) (HIS.H8, cat#: MA1-21315, RRID: AB_557403, Thermo Fisher Scientific, USA) followed by goat anti-mouse IgG horseradish peroxidase enzyme (HRP)-conjugated secondary antibody (1: 10 000) (cat#: 31430, Thermo Fisher Scientific, USA). Proteins were visualised using chemiluminescent substrate (Clarity Western ECL Substrate, Bio-Rad, USA) as previously described [14]. The concentration of purified McsB was determined by the Bradford assay following manufacturer’s instructions (cat #: B6916, Millipore Sigma, USA).

### Thermal shift assay

The thermal shift assay (TSA) was performed by incubating 0.1 µg/µl of purified McsB in assay buffer (50 mM sodium phosphate, 300 mM NaCl, pH 7.4) with 10X SYPRO Orange fluorescent dye (ThermoFisher Scientific, USA) in a final reaction volume of 20 µl [15]. For primary screening, compounds from the Pandemic Response Box were tested in a pooled format (10 compounds per pool at 10 µM each, 40 pools total). Each pool was incubated with the protein-dye mixture for 1 h at 4 °C, followed by thermal denaturation (25–99 °C, at a ramp rate of 0.5 °C/s). Fluorescence was monitored at 470/586 nm (excitation/emission) using a QuantStudio 5 Real-Time PCR System (Applied Biosystems, ThermoFisher Scientific, USA). Compounds from pools that induced a significant increase in McsB melting temperature (T_m_) relative to a DMSO control– indicating engagement with the target protein– were further evaluated individually at 10 µM. All T_m_ values were determined from derivative curves (dF/dT) using Protein Thermal Shift Software v1.4 (ThermoFisher Scientific, USA) and represent the average of at least three independent replicates (*n* ≥ 3).

### Cellular thermal shift assay

CETSA was performed as previously described [16–18]. Briefly, *E. coli* lysate containing McsB was incubated with 1 µM test ligands or DMSO control for 1 h at 4 °C. Each sample was divided into 14 equal aliquots which were subjected to distinct temperatures ranging from 43 °C to 56 °C for 6 min, followed by immediate transfer and incubation on ice for a further 6 min. The aliquots were then centrifuged (10 000 *x g*, 1 h, 4 °C) to pellet insoluble material and the soluble fractions were resolved by SDS-PAGE. Western blot was conducted as described above and protein band intensities were quantified using ImageJ software [19]. Thermal denaturation curves were generated by normalizing band intensities to the highest (100%) and lowest (0%) values for each dataset. The aggregation temperature (T_agg_), representing the temperature at which 50% of the protein aggregates, was determined through nonlinear regression analysis of the normalized data using a Boltzmann sigmoidal model in GraphPad Prism (software version 10.4) [18]. Compounds that increased T_agg_ of McsB relative to the DMSO control– indicating engagement with the protein in a cellular environment– were prioritized for further investigation.

### Isothermal dose-response fingerprint CETSA

Isothermal dose-response fingerprint CETSA (ITDRF_CETSA_) was performed as previously described by Jafari et al. [18]. Soluble McsB protein was incubated with MMV1593539, MMV1580485, MMV1633968 or MMV1782213 (0 (DMSO), 1, 10 or 30 µM) for 1 h at 4 °C, followed by thermal denaturation (48 °C, 6 min) and immediate cooling on ice (6 min). After centrifugation (10 000 *x g*, 4 °C, 1 h), the soluble fraction was resolved by SDS-PAGE and analyzed by western blot as described above. Protein band intensities were quantified using ImageJ [19] and further processed using GraphPad Prism (v10.4).

### Minimum inhibitory concentration assay

The minimum inhibitory concentrations (MIC) of six McsB-targeting ligands (MMV1593539, MMV1578899, MMV1634391, MMV1633968, MMV1782355 and MMV1782213) were determined against wild-type *S. aureus* (ATCC25923.) Overnight cultures grown in 2xYT medium at 37 °C were diluted to OD600 ≈ 0.10 and dispensed into 96-well plates. Compounds were tested in two-fold serial dilutions (20–0.08 µM), with DMSO (vehicle control) and ciprofloxacin (20 to 0.08 µg/ml) included for comparison. Bacterial growth was monitored via OD600 measurements at 0 and 24 h during incubation at 37 or 43℃ (to assess heat shock response) [20]. Growth inhibition was calculated by normalizing ΔOD600 (24 h–0 h) to the DMSO control (set as 100% growth). To evaluate ciprofloxacin potentiation, combination assays using a fixed ligand concentration (20 µM) with serial two-fold dilutions of ciprofloxacin (2.5–0.156 µg/ml, two-fold dilutions) were performed. Bacterial inhibition was quantified after 24-hour incubation at 37℃ as described above.

### Molecular modelling of *S. aureus* McsB

All molecular modelling was performed using Schrödinger computational chemistry software suite (Maestro, version 2022-1) through the Centre for High Performance Computing, Cape Town, South Africa. Three crystal structures were used: the *S. aureus* McsB structure (PDB: 8GQD) [21, 22] and two *Geobacillus stearothermophilus* McsB structures (PDB: 6FH3 and 6FH2) [1, 23, 24]. As the *G. stearothermophilus* structures were crystalized as dimers with clearly resolved pArg or AMP-PN in their binding pockets, we generated a comparable *S. aureus* McsB dimer by removing two monomers from the native tetrameric form [21]. Following protein preparation using the Protein Preparation Wizard (default settings), SiteMap was used to identify five putative binding sites on the *S. aureus* McsB dimer. Using the Protein Structure Alignment tool, we compared these sites with the AMP-PN and pArg binding pockets in *G. stearothermophilus* McsB. One *S. aureus* McsB site aligned with the AMP-PN binding site and another matched the pArg binding site of *G. stearothermophilus* McsB. These sites were then selected for subsequent docking analyses.

### Molecular docking of novel *S. aureus* McsB binders

The six McsB- targeting ligands, MMV1593539, MMV1578899, MMV1634391, MMV1633968, MMV1782355 and MMV1782213, along with AMP-PN, ATP, pArg and Arg were prepared for docking using Schrödinger’s LigPrep tool (default settings). Receptor grids were generated for the predicated ATP and pArg sites of *S. aureus* McsB and for the experimentally resolved AMP-PN- and pArg- binding sites of *G. stearothermophilus* McsB using the Receptor Grid Generation tool. Initial validation docking was performed with Glide (XP mode) where ATP and AMP-PN were docked into the ATP site, while pArg and Arg were docked into pArg sites. Docking scores were compared between species to assess binding site conservation. Thereafter, the six McsB ligands were docked into the *S. aureus* McsB ATP and pArg sites, applying a docking score threshold of -4.3 kcal/mol to prioritize high-affinity interactions.

### Molecular dynamics simulations of *S. aureus* McsB in Apo and ligand-bound form

MD simulations were performed on *S. aureus* McsB monomer in apo state and in complex with ATP, MMV1782355 or MMV1593539 using Desmond (Schrödinger). All systems were simulated for 100 ns under NPT ensemble conditions (300 K, 1.01 bar) with 150 mM NaCl to maintain physiological relevance. To assess system stability and ligand binding characteristics, we analysed RMSD, radius of gyration (Rg) and RMSF. For ligand-bound systems, we additionally tracked interactions and bond persistence throughout the simulations. Snapshots at 20 ns intervals (20, 40, 60, 80 and 100 ns) were used to generate interactions diagrams and evaluate binding mode stability.

### MM-GBSA binding free energy analyses

To quantify binding free energies, MM-GBSA calculations were performed on the MD trajectories of McsB complexed with ATP, MMV1782355 and MMV1593539 using Schrödinger’s Prime MM-GBSA module. Free energy decomposition analysis was conducted at 50-frame intervals across the trajectories to determine ΔG_*bind*_ and its individual components: ΔG_*coulomb*_, ΔG_*covalent*_, Δ*G*_*Hbond*_, ΔG_*lipo*_, ΔG_*packing*_, ΔG_*solvGB*_, and ΔG_*VdW*_ contributions [25]. All energy components were reported as mean ± SD values for each ligand-protein complex.

### Statistical analyses

All statistical analyses were performed using GraphPad Prism (version 10.4). Data were analyzed by either unpaired two-tailed Student’s t-tests or oneway ANOVA followed by Dunnett’s multiple comparison test, as appropriate for each experimental design.

## Results

### Screening of the Pandemic Response Box *via* thermal stability assays

**Fig. 1:**
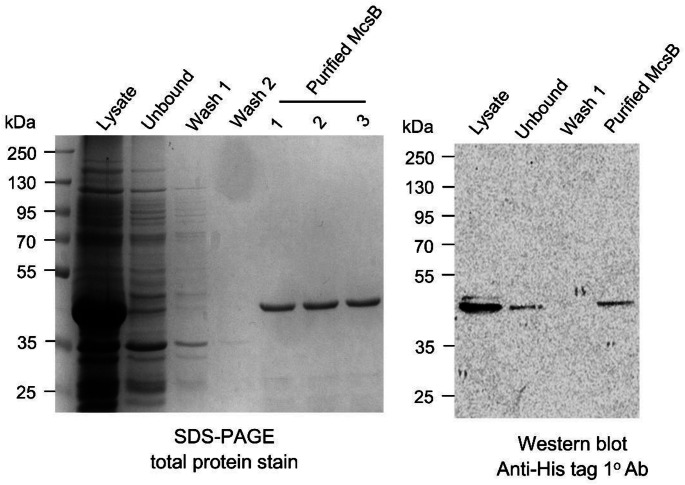
Purification of McsB using Ni^2+^-affinity chromatography. The McsB protein was expressed in *E. coli* Bl21(DE3) cells and purified with Ni-NTA resin. Purification was analysed by SDS-PAGE and western blot using anti-His tag primary antibodies (1^o^ Ab)

**Fig. 2:**
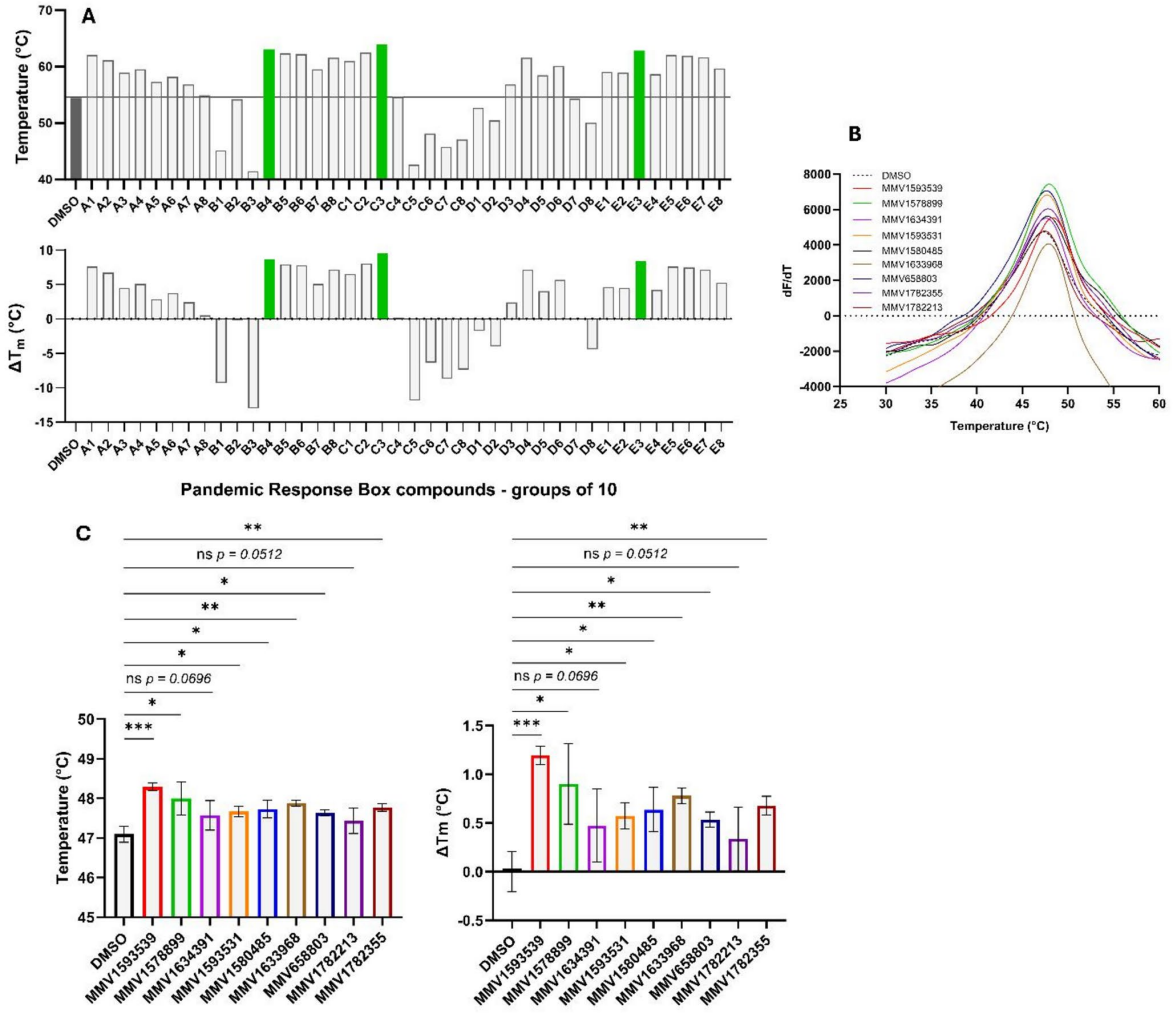
Screening the Pandemic Response Box against McsB using the thermal shift assay (TSA). The protein stability, as represented by T_m_ and ΔT_m_, of the purified McsB protein in the presence of 40 pools of 10 compounds was determined using TSA where green bars represent those pools that were further analysed (**A**). Representative thermal melt profiles (dF/dT) of the nine compounds; MMV1593539, MMV1578899, MMV1634391, MMV1593531, MMV1580485, MMV1633968, MMV658803, MMV1782213 and MMV1782355 (**B**) and their respective mean ± SD T_m_ and ΔT_m_ (*n* = 3) (**C**). Statistical analysis was performed using a student t-test; *p < 0.05, **p < 0.01, ***p < 0.001, ****p < 0.0001, ns: not significant (α = 0.05, CI 95%)

Recombinant McsB was expressed in *E. coli* BL21(DE3) cells and purified using Ni-NTA resin. SDS-PAGE and western blot analysis confirmed the isolation of a ~ 42 kDa His-tagged protein (Fig. 1), consistent with previous reports [1]. The Pandemic Response Box was divided into 40 pools containing 10 compounds each and screened for stabilizing ligands of the purified McsB using TSA. Three groups- B4, C3 and E3- produced the most pronounced increases in McsB melting temperature, elevating it from 54.5 °C to 63.1, 64.0 and 62.8 °C, respectively (Fig. 2A). To identify the active compounds, we screened the 30 individual compounds from these pools, and found nine compounds that increased McsB melting temperature by 0.5–1.2 °C (Fig. 2B and C). Of these, seven induced statistically significant shifts, with MMV1593539 yielding the greatest stabilization (ΔT_m_ = 1.2 ± 0.1 °C, *p* < 0.0001). Notably, the individual compound effects (average ΔT_m_ ≈ 0.7 °C) were substantially less than the pooled group effect (average ΔT_m_ ≈ 8.8 °C), suggesting additive or synergistic interactions among the compounds.

**Fig. 3:**
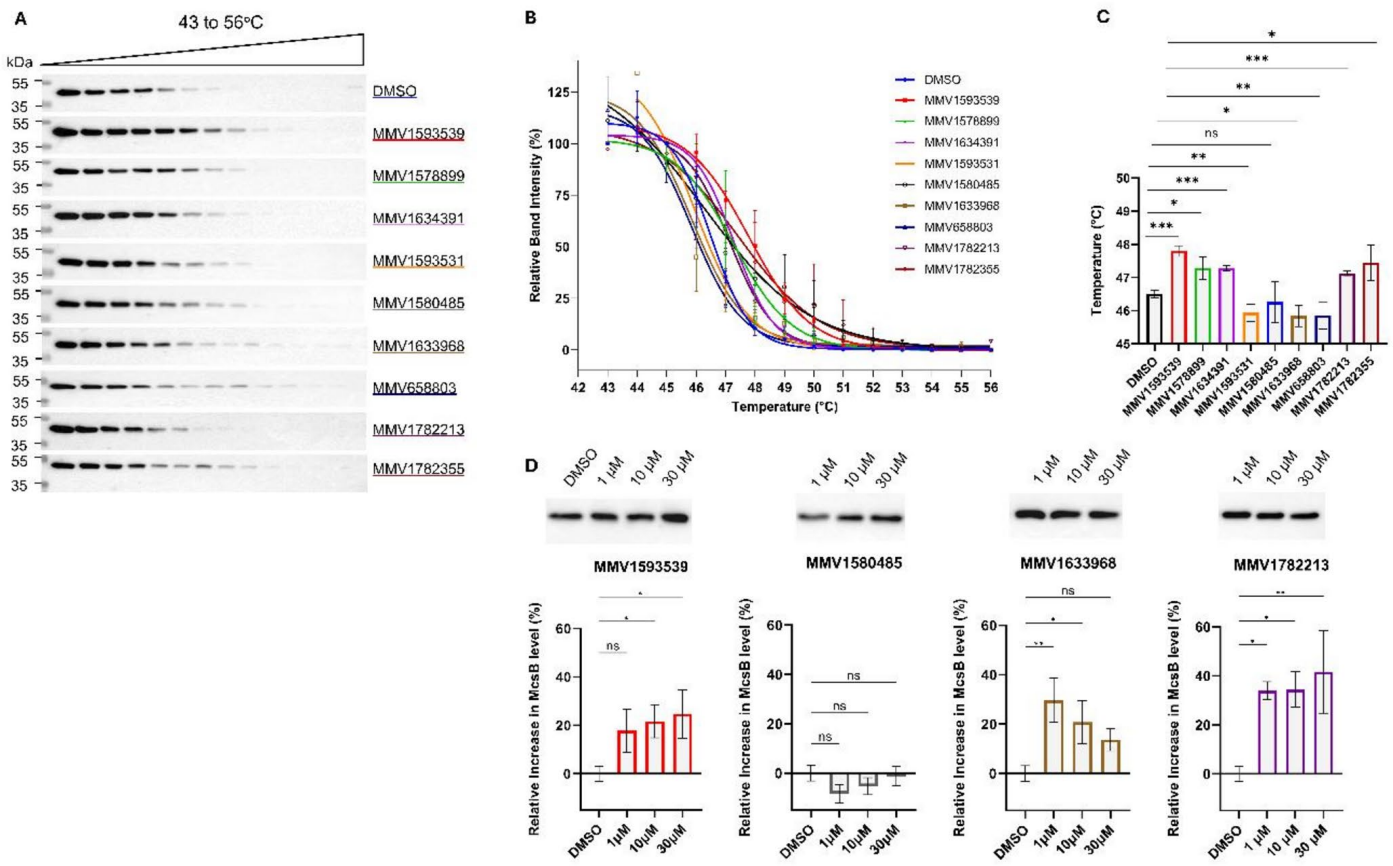
McsB ligand binding through cellular thermal shift assay (CETSA) and isothermal dose-response fingerprint CETSA (ITDRF_CETSA_). The thermal stability of McsB was determined in the presence of 1 µM of each ligand or DMSO control using CETSA by analysing the soluble fraction by western blot with anti-His tag primary antibodies (**A**) with relative band intensity Boltzmann sigmoidal curves (**B**) and mean ± SE aggregation temperatures (T_agg_) (*n* = 3) (**C**). ITDRF_CETSA_ was performed by analysing McsB level at 48 °C in the presence of 0 (DMSO), 1, 10 and 30 µM of MMV1593539, MMV1782213, MMV1580485 or MMV1633968 using western blot with anti-His tag antibodies (**D**). Statistical analysis was performed using a student ttest (**A**-**C**) or oneANOVA with Dunnett’s post hoc (**D**); *p < 0.05, **p < 0.01, ***p < 0.001, ns: not significant (α = 0.05, CI 95%)

To assess target engagement in a native environment, we analyzed the nine candidate ligands using CETSA. Five compounds significantly increased McsB aggregation temperature by 0.63–1.30 °C (Fig. 3A-C), confirming engagement with McsB. Among these, MMV1593539 and MMV1782355 conferred the greatest stabilizing effect (ΔT_agg_ = 1.30 ± 0.16 °C, *p* < 0.001; and 0.95 ± 0.54 °C, *p* ≤ 0.05, respectively), consistent with their TSA results. Interestingly, MMV1634391 and MMV1782213- which had non-significant effects in TSA- significantly stabilized McsB in CETSA (ΔT_agg_ = 0.63 ± 0.08, *p* < 0.001; and 0.78 ± 0.09 °C, *p* < 0.001, respectively), suggesting enhanced interaction in a native environment, possibly mediated by cellular factors. Conversely, MMV1580485 and MMV1633968 stabilized McsB in TSA but not in CETSA, indicating their interactions may be disrupted in a cellular environment.

To further evaluate endogenous binding, we performed ITDRF_CETSA_ on four prioritized compounds. MMV1593539, MMV1633968 and MMV1782213 significantly increased McsB levels after heating at 48 °C across multiple concentrations with dose dependent trends observed for MMV1593539 and MMV1782213 (Fig. 3D). MMV1633968 increased McsB levels by > 20% at 1 and 10 µM, but this effect diminished at higher concentrations, likely due to its poor aqueous solubility (predicted Log *S* = -6.55) [26]. Combined with the TSA results, this demonstrates that MMV1633968 interacts with McsB but does not induce sufficient stabilization to significantly alter its T_agg_. In contrast, MM15480485 yielded no stabilization in ITDRF_CETSA_, consistent with its lack of effect in CETSA.

Collectively, the thermal stability assays identified six compounds that engage with McsB, with MMV1593539 exerting the most pronounced stabilizing effect (Online Resource 1 - Table S1).

### Antibacterial activity

**Fig. 4:**
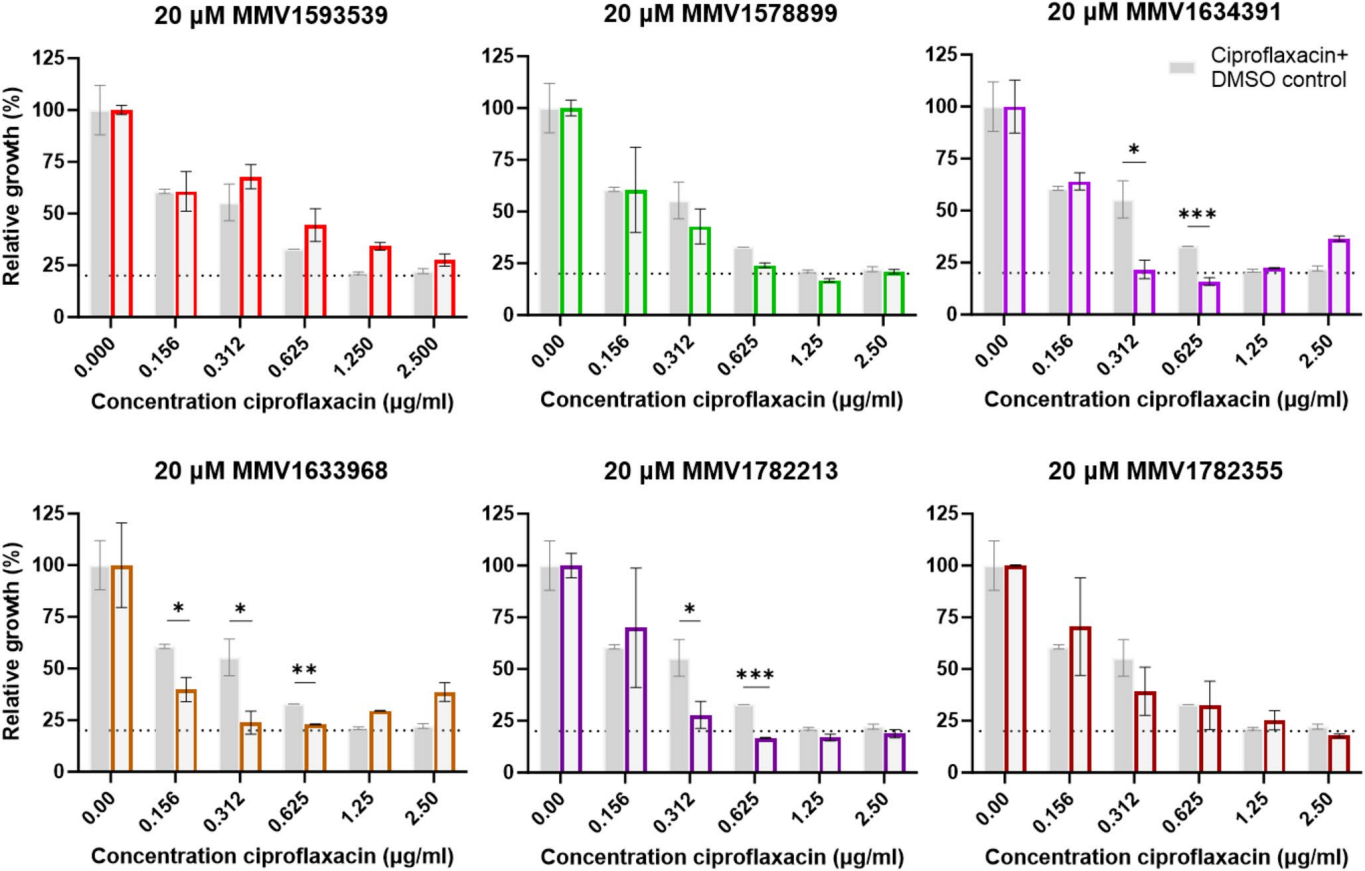
McsB ligands as potentiators of the anti-bacterial activity of ciprofloxacin against *Staphylococcus aureus.* Minimum inhibitory concentration (MIC) assay was done in the presence of ciprofloxacin (0.0–2.5 µg/ml) and 20 µM of either MMV1593539, MMV1578899, MMV1634391, MMV1633968, MMV1782355, MMV1782213 or DMSO only (control) where OD600 was used to determine relative growth of *S. aureus* after 24 h incubation. Statistical analysis was performed using a student t-test; *p < 0.05, ***p < 0.001 (α = 0.05, CI 95%)

McsB is essential to the survival of *S. aureus* with prior studies clearly demonstrating reduced viability of McsB deletion mutants under stress conditions [12]. To evaluate the antibacterial activity of the six McsB targeting ligands, we determined MIC’s against *S. aureus* under standard (37℃) and stress-inducing conditions (43℃, or through addition of ciprofloxacin). None of the compounds - including three classified as anti-bacterial agents within the Pandemic Response Box- inhibited growth of *S. aureus*, under normal or elated temperatures, at the concentrations tested. However, three ligands effectively potentiated ciprofloxacin activity. Of these, MMV1634391 demonstrated the strongest potentiation effect, shifting the MIC80 of ciprofloxacin from 1.25 µg/ml to 0.625 µg/ml (Fig. 4).

### Molecular Docking and dynamics of McsB-ligand interactions

To identify the binding sites of the six McsB-targeting ligands, we conducted molecular modelling. A comparison of the structures of *S. aureus* and *G. stearothermophilus* McsB using SiteMap and Protein Structural Alignment tools allowed for the prediction of putative ATP and pArg sites on *S. aureus* McsB (Online Resource 1 - Figure S1). Both ATP and its non-hydrolyzable analogue AMP-PN docked favourably into putative and known ATP binding sites of *S. aureus* and *G. stearothermophilus* McsB, respectively (Online Resource 1, Table S2). Similarly, docking of pArg and Arg into the putative site on *S. aureus* McsB returned scores comparable to pArg in the known *G. stearothermophilus* binding site (Online Resource 1 - Table S2).

Subsequent docking of the six candidate ligands using Glide revealed preferential binding to the ATP site over the pArg site (Tables 1 and 2). Four compounds exhibited significant affinity for the ATP pocket, with MMV1782355 (-7.873 kcal/mol) and MMV1593539 (-6.518 kcal/mol) returning docking scores comparable to AMP-PN (-7.095 kcal/mol) (Table 2). While MMV17282355 also demonstrated weak binding to the pArg site (-4.486 kcal/mol), two ligands failed to dock effectively into either site– suggesting a potential allosteric binding mechanism.

**Table 1:** Ligand Docking scores for in vitro binders for *Staphylococcus aureus* McsB in the pArg binding site

Ligand	docking score	XP glide	glide emodel
pArg	-5.488	-7.478	-69.504
Arg	-5.094	-5.094	-27.755
MMV1782355	-4.486	-4.52	-49.107

Docking score of >-4.3 used as cut-off

**Table 2:** Ligand Docking scores for in vitro binders of *Staphylococcus aureus* McsB in the ATP binding site

Ligand	docking score	XP glide	glide emodel
ATP	-12.616	-12.616	-95.614
MMV1782355	-7.873	-7.907	-56.45
MMV1782355	-7.272	-7.305	-52.927
AMP-PN	-7.095	-7.095	-52.703
MMV1593539	-6.518	-6.518	-63.139
MMV1634391	-6.114	-6.319	-82.107
MMV1634391	-5.65	-5.854	-79.681
MMV1633968	-5.398	-5.398	-47.67
MMV1633968	-5.093	-5.093	-49.101
MMV1633968	-5.003	-5.003	-49.818

Docking score of >-4.3 used as cut-off

**Fig. 5:**
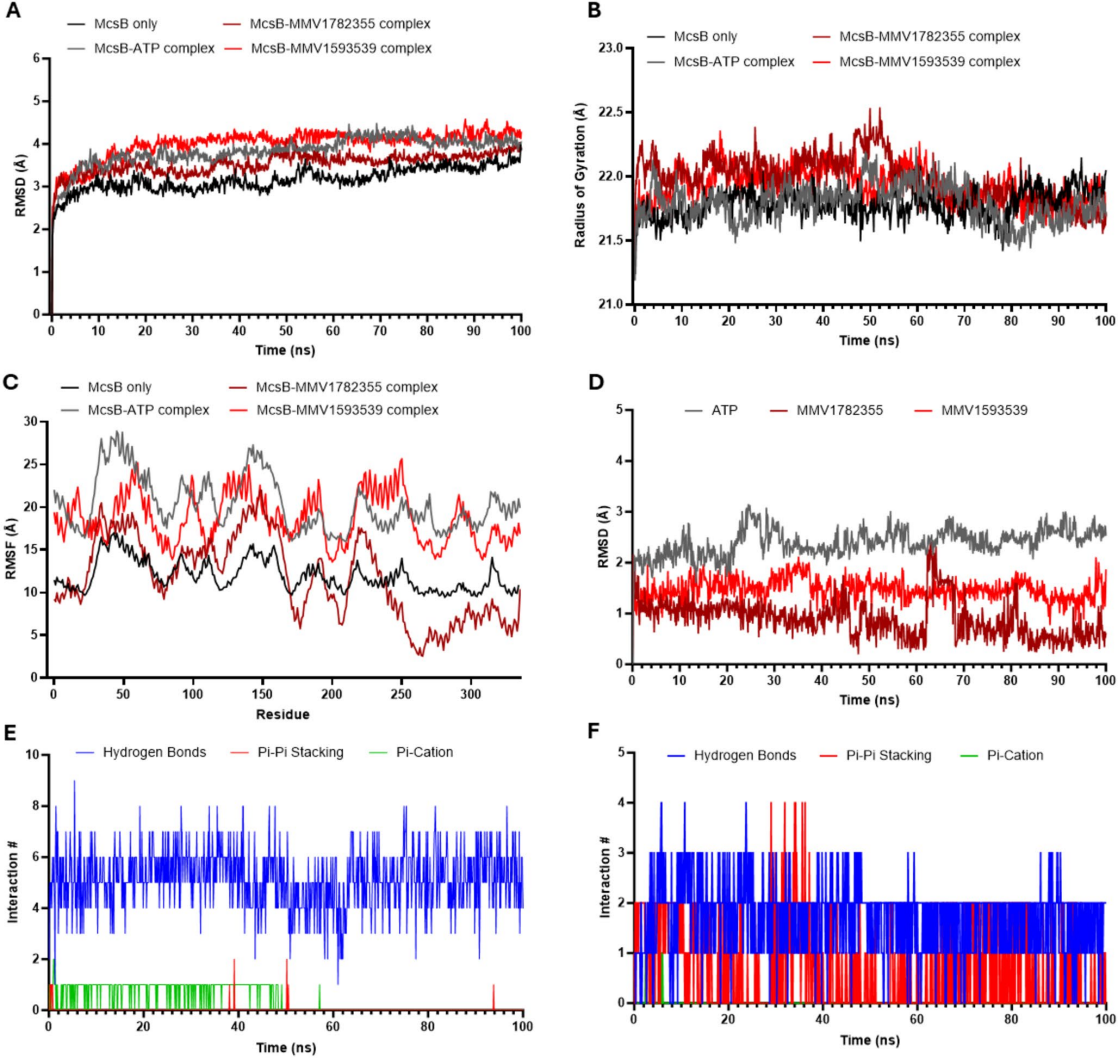
Molecular dynamic (MD) simulation of ATP, MMV1782355 and MMV1593539 in complex with *Staphylococcus aureus* McsB. MD simulations were performed on the *S. aureus* McsB monomer for 100 ns with no ligand or in complex with ATP, MMV1782355 and MMV1593539 using Desmond with root mean square deviation (RMSD) over time (**A**), radius of gyration (Rg) over time (**B**) and root mean square fluctuation (RMSF) per residue (**C**). RMSD of ATP, MMV1782355 and MMV1593539 only over time (**F**) with bond interaction counts for MMV1782355 (**E**) and MMV1593539 (**F**) with McsB are also shown

To characterize the binding dynamics of MMV1782355 and MMV1593539 with McsB, we performed MD simulations comparing ligand-bound complexes with both the apo protein and the ATP-bound protein as references. All systems achieved stable structural formation from ~ 10 ns (Fig. 5A). The average RMSD values over the 10–100 ns window were 3.214 ± 0.23 for apo McsB, 3.911 ± 0.22 Å for the ATP-bound complex, 3.588 ± 0.18 Å for MMV1782355-bound complex and 4.095 ± 0.17 Å for MMV1593539-bound complex (Fig. 5A, Online Resource 1 - Table S3). These values, all within 1–2 Å of the apo structure, confirm stable binding [27]. Although the RMSD of the MMV159539-bound complex continued to rise until 20 ns, it exhibited the least fluctuation of any complex thereafter (Online Resource 1– Table S3), indicating enhanced stability after initial conformational adjustments.

Radius of gyration measurements revealed minimal structural perturbations across all systems during the 10–100 ns simulation window. The average Rg values were consistent at 21.8 ± 0.11 Å for apo McsB, 21.8 ± 0.14 Å for ATP-bound complex, 22.0 ± 0.18 Å for MMV1782355-bound complex and 21.9 ± 0.13 Å for MMV1593539-bound complex (Fig. 5B, Online Resource 1 - Table S3) indicating that ligand binding did not significantly alter the overall compactness of the protein. RMSF analysis revealed distinct dynamic patterns between ligand-bound and apo states. While the apo protein maintained relatively rigid conformation, all three ligand-bound complexes experienced increased residue flexibility (Fig. 5C), consistent with localized structural dynamics induced by protein-ligand interactions.

We then used our MD simulations to evaluate the key binding interactions of MMV1782355 and MMV1593539 with McsB. The ligand RMSD analysis revealed greater fluctuations for both ATP and MMV1782355 whereas MMV1593539 had little fluctuation over the 100 ns (Fig. 5D, Online Resource 1 - Table S3). Throughout the simulation, MMV1782355 formed substantially more hydrogen bonds (H-bonds) with McsB (average: 5.2) compared to MMV1593539 (average: 1.7) (Fig. 5E and F). A persistent H-bond between MMV1782355 and Glu102 was observed throughout the simulation, while additional transient H-bonds involved the pyridine and guanidine-like rings with residues Trp12, Asp7 and Lys81 (20–40 ns), Ser24 and Lys81 (60 ns), and Asp8 and Gln309 (80–100 ns). A single Pi-cation interaction was maintained for 50 ns (Fig. 5E, Online Resource 1– Figure S2). In contrast, MMV1593539 exhibited more consistent interactions, including sustained Pi-Pi stacking with His82 and an H-bond with Glu102 over the entire 100 ns simulation. Additional stabilizing interactions included a second Pi-Pi stacking with Typ12, an H-bond with Ser105/Gln117, and a Pi-cation bond with Lys81 (Online Resource 1– Figure S2). These findings were supported by Post-MD MM-GBSA analysis which revealed that MMV1593539 had more favourable total binding free energy (ΔG_bind_ = -53.460 ± 6.13 kcal/mol) than MMV1782355 (ΔG_bind_ = -33.935 ± 4.89 kcal/mol), closely resembling that of ATP (ΔG_bind_ = -60.405 ± 9.96 kcal/mol) (Table 3). The favourable total free-binding energy of MMV1593539 was driven by ΔG_packing_ and ΔG_VdW_ contributions, highlighting their importance to stable complex formation.

While molecular docking predicted stronger binding for MMV1782355, MD simulations and post-MD analysis demonstrated that MMV1593539 formed a more stable and consistent complex with McsB. These results align well with our in vitro observations, where MMV1593539 conferred the greatest thermal stability to McsB.

**Table 3:** The MMGBSA predicted total binding free energies and energy components of ATP, MMV1782355 and MMV1593539

	ATP	MMV1782355	MMV1593539
ΔG_coulomb_^a^ (kcal/mol)	-49.690 ± 11.29	-19.239 ± 3.94	-9.704 ± 5.49
ΔG_covalent_^b^ (kcal/mol)	1.385 ± 2.47	0.986 ± 0.51	1.081 ± 0.75
ΔG_Hbond_^c^ (kcal/mol)	-3.728 ± 0.89	-2.165 ± 0.65	-1.200 ± 0.33
ΔG_lipo_^d^ (kcal/mol)	-7.506 ± 1.06	-8.401 ± 1.07	-12.779 ± 0.76
ΔG_packing_^e^ (kcal/mol)	-3.721 ± 3.40	-1.580 ± 1.22	-4.610 ± 1.05
ΔG_solvGB_^f^ (kcal/mol)	50.365 ± 6.24	32.199 ± 3.24	25.899 ± 4.33
ΔG_VdW_^g^ (kcal/mol)	-47.505 ± 7.21	-35.733 ± 3.09	-52.148 ± 2.06
ΔG_bind_^h^ (kcal/mol)	-60.405 ± 9.96	-33.935 ± 4.89	-53.460 ± 6.13

^a^Contribution of coulomb energy to the binding free energy

^b^Covalent-bonding contribution to the binding free energy

^c^Hydrogen-bonding contribution to the binding free energy

^d^Lipophilic energy contribution to the binding free energy

^e^Pi-Pi packing energy contribution to the binding free energy

^f^Generalized Born electrostatic solvation energy contribution to the binding free energy

^g^Contribution of Van der Waals interaction energy to the binding free energy

^h^Total binding free energy

## Discussion

Screening of the Pandemic Response Box for McsB ligands returned six compounds of interest. Three of these compounds; MMV1593539, MMV1578899 and MMV1634391, were originally categorized as antibacterial agents in the Pandemic Response Box. In previous work, the bis-indole, MMV1593539, was found to inhibit the growth of organisms such as *Haemonchus contortus* and *Plasmodium falciparum* as well as the spore forming capabilities of *Nematocida parisii* and *Pancytospora epiphaga* through screens of the Pandemic Response Box [28–30]. The antibactericidal property of MMV1593539 stems from its marine natural product-inspired design as a selective inhibitor of methicillin-resistant *S. aureus* pyruvate kinase (PK) activity [31–33] - even though it was found ineffective against *S. aureus* strains when tested at single, high, range-finding doses [31, 34]. As this compound is only moderately soluble and similar to a molecule previously found to aggregate [35], we opted to screen a dose range encompassing concentrations lower than previously tested in literature. No improvement in activity was evident however, with marginal growth inhibition observed only at the highest concentration tested. Structural studies have clarified the interaction of certain bis-indoles, such as indolocarbazoles and bis-indolyl maleimides, with ATP binding pockets [36, 37]. In these cases, the γ-lactam and maleimide groups provided important H-acceptor and donor groups to stabilize the interaction. In our models, the unsubstituted bis-indole MMV1593539 bound into the ATP binding pocket, albeit with less affinity than ATP. Taken together, MMV1593539 presents a useful starting point as a ligand of the ATP binding site of McsB.

While MMV1593539 offers the clearest ligand potential, MMV1578899 warrants further consideration as an McsB ligand. This fragment-like compound, which has not been highlighted as a hit molecule from any previous reported screen of the Pandemic Response Box, engaged recombinantly expressed McsB directly and within a cellular environment and effectively stabilized the protein on both accounts. Accordingly, MMV1578899 likely forms specific, potentially exclusive, high-quality interactions with McsB. Not unexpectedly, the fragment did not affect growth of *S. aureus* over the concentration tested within our assay. The antibacterial classification of this compound seemingly stems from its potentiation of the anti-*M. tuberculosis* activity of ethionamide [38] with no growth inhibition activity reported at single, high-dose, evaluations [39]. Through fragment building, affinity of the interaction with McsB can be increased to reveal the ligand binding site/s and anti-bacterial potential of this molecule.

Our study reported little effect on *S. aureus* growth in the presence of MMV1634391. MMV1634391 (MGB-BP-3), a minor groove binder, has been reported to inhibit the growth of several gram-positive bacteria including *S. aureus* with a reported MIC80 ~ 0.1 µM [40, 41]. Notably, MMV1634391 did not induce a significant increase in McsB T_m_ but did increase McsB T_agg_. Since we were only able to observe a shift in McsB T_agg_ in the endogenous environment offered in the CETSA, this may suggest binding in the presence of preformed complexes or the need for certain cofactors found inside the bacterial cell. Some gram-negative bacteria, specifically those from the phylum Proteobacteria in which *E. coli* is classed, possess homologues of McsA and McsB [42]. Moreover, McsB was recently reported to bind in transient to the CtsR-DNA complex [43]. Therefore, if the role of McsB in *E. coli* and *S. aureus* are similar, it may be plausible that the entire McsB-CtsR (or equivalent *E. coli* repressor)-DNA-MMV1634391 complex was stabilised in the endogenous environment. Although no homolog of CtsR has been reported for *E. coli*, similar heat shock repressors exists [44]. Additional research is required to determine the exact binding mechanism of this compound and whether this interaction with McsB contributes to the antibacterial effect often observed against gram-positive bacteria.

Three compounds classified as anti-viral agents in the Pandemic Response Box were also identified as McsB ligands in this study. MMV1633968 (OBR-5-340), a broad-spectrum rhinovirus capsid formation inhibitor [45] and MMV1782355 (peldesine or BCX-34), a potent purine nucleoside phosphorylase inhibitor [46], engaged McsB in the both the TSA and CETSA. MMV1782355 returned the most favourable docking scores of all compounds screened and stabilized cellular McsB more convincingly than MMV163398. Interestingly, both molecules possess purine-like rings suggesting a comparable binding mechanism and a structural requirement for interaction, with preference for guanine rings over adenine rings.

The anti-viral agent, MMV1782213 (RDEA806), a nonnucleoside reverse transcriptase inhibitor– active against HIV– was previously shown to be inactive against methicillin-resistant *S. aureus* [39], similar to in our study. Like MMV1634391, MMV1782213 also induced better thermal stability of McsB in the CETSA but we were unable to find favourable docking poses in the pArg and ATP sites, suggesting that this compound may be binding allosterically, possibly at a protein-protein interface. Future studies should focus on determining whether the binding of MMV1782213 to McsB inhibits the enzyme. Since MMV1782213 is likely binding outside the two essential active sites of McsB, we propose using both in vitro and in vivo activity assays– to ensure any protein-protein interfaces remain intact– similar to those outline by Suskiewics et al. [1] to assess the activity of this McsB binder.

As a requisite component of the bacterial stress signalling and protein quality control systems, McsB is a potential target for antimicrobial therapy. The exploitation of this target is restricted by the absence of small molecule ligands and inhibitors. The six compounds identified in this study represent, to the best of our knowledge, the first reported ligands of McsB. Of these, the four active-site binding ligands present viable scaffolds for classic inhibitors. Optimization and refinement of these ligands would, however, be necessary in order to achieve appreciable McsB inhibition and impact bacterial viability. Over the last two decades, a novel drug discovery strategy has arisen in which disease-related proteins are targeted for removal by co-opting endogenous proteolytic systems [40, 41]. Binding McsB, a protein akin to the E3 ligases of eukaryotes, could open new avenues for potential degrader / BacPROTAC development. It would be intriguing to assess whether degraders formulated with our ligands would have the propensity to degrade a target protein. In principle, the recruitment of McsB to a target protein would promote phosphorylation of the target protein, labelling it for ClpCP proteasomal degradation. ClpCP itself has previously been co-opted in proof-of-concept studies, where target proteins were successfully removed [47, 48]. Two of the ligands identified in this study, MMV1578899 and MMV1782213, bind outside the pArg and ATP pockets and thus would not directly impair the labelling function of McsB. We therefore propose these two molecules as possible starting points to develop novel BacPROTACs– provided their binding site/s are delineated through future studies. In conclusion, screening of the Pandemic Response Box identified six novel ligands which represent promising candidates for future drug discovery efforts aimed at exploiting the protein arginine kinase, McsB.

## Electronic supplementary material

Below is the link to the electronic supplementary material.

Supplementary Material 1

## Data Availability

No datasets were generated or analysed during the current study.

## Abbreviations

CETSA: Cellular thermal shift assay
TSA: Thermal shift assay
