# Supplementary material for: Screening the Pandemic Response Box identifies novel ligands of the Staphylococcus aureus protein arginine kinase, McsB
Source: Mol Biol Rep. 2025 May 6;52(1):446. doi: 10.1007/s11033-025-10545-9 (PMC12055656; doi:10.1007/s11033-025-10545-9)
Supplement: Supplementary file 1 — Supplementary Material 1 [file 11033_2025_10545_MOESM1_ESM.pdf]

Online Resource 1

Title Page

Title: Screening the Pandemic Response Box identifies novel ligands of the *Staphylococcus aureus* protein arginine kinase, McsB

Author Affiliation and ORCID numbers:

Ryan Chetty[1] (0000-0002-9280-1846) - ORCID, Alexandré Delport[1] (0000-0003-3511-4369) - ORCID, Clinton Veale[2] (0000-0002-4043-7106) - ORCID and Raymond Hewer[1] (0000-0002-6025-8826) - ORCID

[1] Discipline of Biochemistry, School of Life Sciences, University of KwaZulu-Natal, Pietermaritzburg 3201, South Africa.

[2] Department of Chemistry, University of Cape Town, Rondebosch, Cape Town 7701, South Africa.

Corresponding Author:

Raymond Hewer[1] Email Address: hewerr@ukzn.ac.za

Clinton Veale[2] Email Address: clinton.veale@uct.ac.za

Supplementary Figure S1: Structural alignment of *Staphylococcus aureus* and *Geobacillus stearothermophilus* McsB. Dimeric *S. aureus* (PDB: 8GQD, two monomers were removed from the tetramer) and dimeric *G. stearothermophilus* (PDB: 6FH3) were prepared using the protein preparation wizard with default setting in the Schrödinger drug discovering suite (Maestro, version 2022-1). The structural alignment tool was used to align all residue from both structures. *Alignment starts on next page*

Structural alignment:

```
                                          ..........+.........+.........+.........
6FH3 - Gs McsB  SSA 1610    4 -HHHHHHCCCHHHHHCCCCCCEEEEEEEEEEEEEECCCCC
8GQD - Sa McsB  SSA 1611    1 C---------------------------------------
6FH3 - Gs McsB      1610    4 -GKFFNTAVSAWMSQEGPNSDIVLSSRIRLARNIVDFRFP
8GQD - Sa McsB      1611    1 M---------------------------------------

                              ..........+.........+.........+.........
6FH3 - Gs McsB  SSA 1610   43 CCCCHHHHHHHHHHHHHHCCCCCCCCCCCCEEEEECCCCH
8GQD - Sa McsB  SSA 1611      ----------------------------------------
6FH3 - Gs McsB      1610   43 TLFSSEEAKQIVALFERAFVHRPYGEAGRFELLKMSELQP
8GQD - Sa McsB      1611      ----------------------------------------

                              ..........+.........+.........+.........
6FH3 - Gs McsB  SSA 1610   83 HHHHHHHHHCCCCCCCCCCCCCEEEEEECCCEEEEEECCC
8GQD - Sa McsB  SSA 1611      ----------------------------------------
6FH3 - Gs McsB      1610   83 IEKRVLVEKHLISPHLAEDSPFGACLLSENEEISIMINEE
8GQD - Sa McsB      1611      ----------------------------------------

                              ..........+.........+.........+.........
6FH3 - Gs McsB  SSA 1610  123 CEEEEEEEECCCCHHHHHHHHHHHHHHHHHCCCCCCCCCC
8GQD - Sa McsB  SSA 1611      ----------------------------------------
6FH3 - Gs McsB      1610  123 DHIRIQCLFPGLQLAEALEAASELDDWIEGHVNYAFDERL
8GQD - Sa McsB      1611      ----------------------------------------

                              ..........+.........+.........+.........
6FH3 - Gs McsB  SSA 1610  163 CCCCCCCCCCECEEEEEEEEECHHHHHHCHHHHHHHHHHH
8GQD - Sa McsB  SSA 1611      ----------------------------------------
6FH3 - Gs McsB      1610  163 GYLTSCPTNVGTGLRASVMMHLPALVLTQQINRIIPAINQ
8GQD - Sa McsB      1611      ----------------------------------------

                              ..........+.........+.........+.........
6FH3 - Gs McsB  SSA 1610  203 HCCEEECCCCCCCCCCCEEEEEEECCCCCCCHHHHHHHHH
8GQD - Sa McsB  SSA 1611      ----------------------------------------
6FH3 - Gs McsB      1610  203 LGLVVRGTYGEGSEALGNIFQISNQITLGKSEEDIVADLH
8GQD - Sa McsB      1611      ----------------------------------------
```

```
                                   ..........+.........+.........+.........
6FH3 - Gs McsB  SSA 1610  243 HHHHHHHHHHHHHHHHHHHHHCHHHHHHHHHHHHHHHCCC
8GQD - Sa McsB  SSA 1611      ----------------------------------------
6FH3 - Gs McsB      1610  243 TIVEQLIAQERAARQALVKTLGIQLEDKVFRSYGILANCR
8GQD - Sa McsB      1611      ----------------------------------------

                              ..........+.........+.........+.........
6FH3 - Gs McsB  SSA 1610  283 CCCHHHHHHHHHHHHHHHHHCCCCCCCHHHHHHHHHHHHH
8GQD - Sa McsB  SSA 1611      ----------------------------------------
6FH3 - Gs McsB      1610  283 VIDSKEAAQCLSDVRLGIDLGYIKNVSRNILNELMILTQP
8GQD - Sa McsB      1611      ----------------------------------------

                              ..........+.........+.........+.........
6FH3 - Gs McsB  SSA 1610  323 CHHHHHHCCCCCHHHHHHHHHHHHHHHHHHHHCCCCCCCC
8GQD - Sa McsB  SSA 1611    2 -----------------------------------CCCCC
6FH3 - Gs McsB      1610  323 GFLQQYAGGVLRPEERDVRRAALIRERLRMETRKFFNTAV
8GQD - Sa McsB      1611    2 -----------------------------------THNIH

                              ..........+.........+.........+.........
6FH3 - Gs McsB  SSA 1610   12 -CHH-HHHCC-CCCCEEEEEEEEEEEEEECCCCCCC-CH-
8GQD - Sa McsB  SSA 1611    7 CCCCHHHHHCCCC-CEEEEEEEEECEEEEC----CCCCCC
6FH3 - Gs McsB      1610   12 -SAW-MSQEG-PNSDIVLSSRIRLARNIVDFRFPTL-FS-
8GQD - Sa McsB      1611    7 DNISQWMKSNEET-PIVMSSRIRLARNLEN----HVHPLM

                              ..........+.........+.........+.........
6FH3 - Gs McsB  SSA 1610   47 ---HHHHHHHHHHHHHHCCCCCEEEEECCCCHHHHHHHHH
8GQD - Sa McsB  SSA 1611   42 CCCHHHHHHHHHHHHHHCCC-CEEEEECCCCHHHHHHHHH
6FH3 - Gs McsB      1610   47 ---SEEAKQIVALFERAFVGRFELLKMSELQPIEKRVLVE
8GQD - Sa McsB      1611   42 YATENDGFRVINEVQDALPN-FELMRLDQMDQQSKMKMVA

                              ..........+.........+.........+.........
6FH3 - Gs McsB  SSA 1610   91 HCCCCHHHHHHCCCEEEEEECCCEEEEEECCC-CEEEEEE
8GQD - Sa McsB  SSA 1611   81 HCCCCHHHHHH-HCEEEEECCCCEEEEEECCCCC-EEEEE
6FH3 - Gs McsB      1610   91 KHLISPHLAEDSPFGACLLSENEEISIMINEE-DHIRIQC
8GQD - Sa McsB      1611   81 KHLISPELIKQ-PAAAVLVNDDESLSVMINEEDH-IRIQA
```

```
                                    ..........+.........+.........+.........
6FH3 - Gs McsB  SSA 1610  130 EECCCC-HHHHHHHHHHHHHHHHHCCCCCCECCCEEECCC
8GQD - Sa McsB  SSA 1611  119 EE-CCCCHHHHHHHHHHHHHCCCCCCECCCCCCCCCCC-E
6FH3 - Gs McsB      1610  130 LFPGLQ-LAEALEAASELDDWIEGHVNYAFDERLGYLTSC
8GQD - Sa McsB      1611  119 MG-TDTTLQALYNQASSIDDELDRSLDISYDEQLGYLT-T

                                    ..........+.........+.........+.........
6FH3 - Gs McsB  SSA 1610  169 C-CCCCCEEEEEEEEECHHHHHHCCHHHHHHHHHHHEEEE
8GQD - Sa McsB  SSA 1611  157 CCEECCCCCEEEEEEECHHHHHHCCHHHHHHHHHHHCCEE
6FH3 - Gs McsB      1610  169 P-TNVGTGLRASVMMHLPALVLTQQINRIIPAINQLGLVV
8GQD - Sa McsB      1611  157 CPTNIGTGMRASVMLHLPGLSIMKRMTRIAQTINRFGYTI

                                    ..........+.........+.........+.........
6FH3 - Gs McsB  SSA 1610  208 ECC-CCCCCCCCCE-EEEEEECCCCCCCHHHHHHHHHHHH
8GQD - Sa McsB  SSA 1611  197 ECCCCC-C-CCCCCCCEEEEECCCCCCCHHHHHHHHHHHH
6FH3 - Gs McsB      1610  208 RGT-YGEGSEALGN-IFQISNQITLGKSEEDIVADLHTIV
8GQD - Sa McsB      1611  197 RGIYGE-G-SQVYGHTYQVSNQLTLGKSELEIIETLTEVV

                                    ..........+.........+.........+.........
6FH3 - Gs McsB  SSA 1610  246 HHHHHHHHHHHHHHHHHHCHHHHHHHHHHHHHHHCCCCCC
8GQD - Sa McsB  SSA 1611  235 HHHHHHHHHHHHHHHHHHCHHHHHHHHHHHHHHHCCCCCH
6FH3 - Gs McsB      1610  246 EQLIAQERAARQALVKTLGIQLEDKVFRSYGILANCRVID
8GQD - Sa McsB      1611  235 NQIIHEEKQIRQKLDTYNQLETQDRVFRSLGILQNCRMIT

                                    ..........+.........+.........+.........
6FH3 - Gs McsB  SSA 1610  286 HHHHHHHHHHHHHHHHHCCCCCCCHHHHHHHHHHHCHHHH
8GQD - Sa McsB  SSA 1611  275 HHHHHHHHHHHHHHHHHCCCC-CCHHHHHHHHHHHC---C
6FH3 - Gs McsB      1610  286 SKEAAQCLSDVRLGIDLGYIKNVSRNILNELMILTQPGFL
8GQD - Sa McsB      1611  275 MEEASYRLSEVKLGIDLNYIE-LQNFKFNELMVAIQ---S

                                    ..........+.........+.........+.........
6FH3 - Gs McsB  SSA 1610  326 HHHHCCCCCHHHHHHHHHHHHHHHHHHHHH----------
8GQD - Sa McsB  SSA 1611  311 CCCCCC-CC---HHHHHHHHHHHHHHHHC-CCCCCCCCCC
6FH3 - Gs McsB      1610  326 QQYAGGVLRPEERDVRRAALIRERLRMETR----------
8GQD - Sa McsB      1611  311 PFLLDE-ED---DKSVKEKRADILREHIK-MTHNIHDNIS
```

```
                                    ..........+.........+.........+.........
6FH3 - Gs McsB  SSA 1610            ----------------------------------------
8GQD - Sa McsB  SSA 1611   11 HHHHHCCCCCEEEEEEEEECEEEECCCCCCCCCCHHHHHH
6FH3 - Gs McsB      1610            ----------------------------------------
8GQD - Sa McsB      1611   11 QWMKSNEETPIVMSSRIRLARNLENHVHPLMYATENDGFR

                                    ..........+.........+.........+.........
6FH3 - Gs McsB  SSA 1610            ----------------------------------------
8GQD - Sa McsB  SSA 1611   51 HHHHHHHHCCCCEEEEECCCCHHHHHHHHHHCCCCHHHHH
6FH3 - Gs McsB      1610            ----------------------------------------
8GQD - Sa McsB      1611   51 VINEVQDALPNFELMRLDQMDQQSKMKMVAKHLISPELIK

                                    ..........+.........+.........+.........
6FH3 - Gs McsB  SSA 1610            ----------------------------------------
8GQD - Sa McsB  SSA 1611   91 HHCEEEEECCCCEEEEEECCCCCEEEEEEECCCCHHHHHH
6FH3 - Gs McsB      1610            ----------------------------------------
8GQD - Sa McsB      1611   91 QPAAAVLVNDDESLSVMINEEDHIRIQAMGTDTTLQALYN

                                    ..........+.........+.........+.........
6FH3 - Gs McsB  SSA 1610            ----------------------------------------
8GQD - Sa McsB  SSA 1611  131 HHHHHHHCCCCCCECCCCCCCCCCCECCEECCCCCEEEEE
6FH3 - Gs McsB      1610            ----------------------------------------
8GQD - Sa McsB      1611  131 QASSIDDELDRSLDISYDEQLGYLTTCPTNIGTGMRASVM

                                    ..........+.........+.........+.........
6FH3 - Gs McsB  SSA 1610            ----------------------------------------
8GQD - Sa McsB  SSA 1611  171 EECHHHHHHCCHHHHHHHHHHHCCEEECCCCCCCCCCCCC
6FH3 - Gs McsB      1610            ----------------------------------------
8GQD - Sa McsB      1611  171 LHLPGLSIMKRMTRIAQTINRFGYTIRGIYGEGSQVYGHT

                                    ..........+.........+.........+.........
6FH3 - Gs McsB  SSA 1610            ----------------------------------------
8GQD - Sa McsB  SSA 1611  211 EEEEECCCCCCCHHHHHHHHHHHHHHHHHHHHHHHHHHHH
6FH3 - Gs McsB      1610            ----------------------------------------
8GQD - Sa McsB      1611  211 YQVSNQLTLGKSELEIIETLTEVVNQIIHEEKQIRQKLDT
```

```
                                        ..........+.........+.........+.........
6FH3 - Gs McsB  SSA 1610                ----------------------------------------
8GQD - Sa McsB  SSA 1611  251 HHCHHHHHHHHHHHHHHHHCCCCCHHHHHHHHHHHHHHHH
6FH3 - Gs McsB      1610                ----------------------------------------
8GQD - Sa McsB      1611  251 YNQLETQDRVFRSLGILQNCRMITMEEASYRLSEVKLGID

                                        ..........+.........+.........+.........
6FH3 - Gs McsB  SSA 1610                ----------------------------------------
8GQD - Sa McsB  SSA 1611  291 HCCCCCCCCCHHHHHHHHHCCCCCCCCCHHHHHHHHHHHH
6FH3 - Gs McsB      1610                ----------------------------------------
8GQD - Sa McsB      1611  291 LNYIELQNFKFNELMVAIQSPFLLDEEDDKSVKEKRADIL

                                        ..........+.........+.........+.........
6FH3 - Gs McsB  SSA 1610                -----
8GQD - Sa McsB  SSA 1611  331 HHHHC
6FH3 - Gs McsB      1610                -----
8GQD - Sa McsB      1611  331 REHIK
```

Alignment Score: 0.336

RMSD: 2.825 Angstrom

Supplementary Table S1: The six *Staphylococcus aureus* McsB ligands identified from the Pandemic Response Box.

MMV number	Structure	IUPAC Name	Drug Class	ΔTm	ΔTagg
MMV1593539	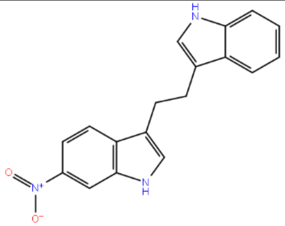	3-[2-(1H-indol-3-yl)ethyl]-6-nitro-1H-indole	Anti-bacterial	1.19 ± 0.095°C	1.3 ± 0.161°C
MMV1578899	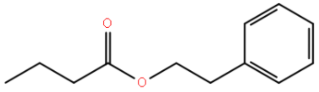	2-phenylethyl butanoate	Anti-bacterial	0.900 ± 0.412°C	0.78 ± 0.339°C
MMV1634391	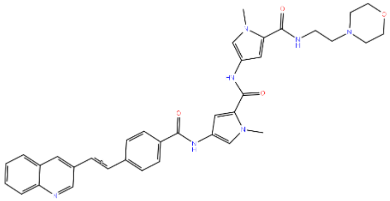	1-methyl-N-[1-methyl-5-(2-morpholin-4-ylethylcarbamoyl)pyrrol-3-yl]-4-[[4-[(E)-2-quinolin-3-ylethenyl]benzoyl]amino]pyrrole-2-carboxamide	Anti-bacterial	0.473 ± 0.374°C	0.78 ± 0.09°C
MMV1633968	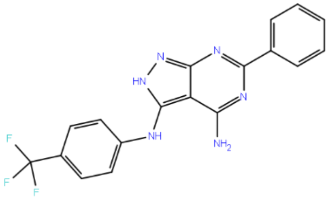	6-phenyl-3-N-[4-(trifluoromethyl)phenyl]-1H-pyrazolo[3,4-d]pyrimidine-3,4-diamine	Anti-viral	0.780 ± 0.081°C	ns[a]
MMV1782355	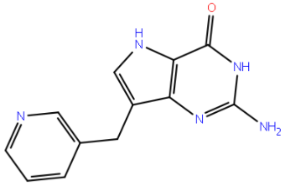	2-amino-7-(pyridin-3-ylmethyl)-3,5-dihydropyrrolo[3,2-d]pyrimidin-4-one	Anti-viral	0.677 ± 0.098°C	0.63 ± 0.54°C

MMV1782213	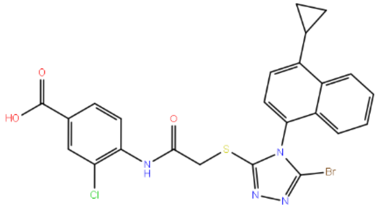	4-[[2-[[5-bromo-4-(4-cyclopropylnaphthalen-1-yl)-1,2,4-triazol-3-yl]sulfanyl]acetyl]amino]-3-chlorobenzoic acid	Anti-viral	0.337 ± 0.323°C	0.95 ± 0.076°C

[a]Selected as a ligand after isothermal dose-response fingerprint produced a significant increase in McsB levels of 20-30% at 1 and 10 µM treatment.

Supplementary Table S2: ***Staphylococcus aureus*** **and** ***Geobacillus stearothermophilus*** **McsB protein-ligand docking score comparison**

McsB Species	Ligand			
	AMP-PN	ATP	pArg	Arg
	Docking scores (kcal/mol)			
S. aureus	-7.095	-12.616	-5.488	-5.094
G. stearothermophilus	-10.230	-12.230	-5.018	-7.383

Supplementary Table S3: ***Staphylococcus aureus*** **McsB average RMSD, Radius of Gyration and RMSF**

	Complex			
**Ligand**	**McsB**	**McsB-ATP**	**McsB-MMV1782355**	**McsB-MMV1593539**
	**RMSD: 10-100 ns for Protein-Ligand complex**			
**Mean ± SD**	3.214 ± 0.23	3.911 ± 0.22	3.588 ± 0.18	4.095 ± 0.17
**Difference**[a]	0	0.697	0.374	0.881
**Min-Max Range**	1.214	1.170	0.9705	1.183
	**RMSD: 20-100 ns for Protein-Ligand complex**			
**Mean ± SD**	3.236 ± 0.23	3.946 ± 0.20	3.608 ± 0.18	4.135 ± 0.12
**Difference**[a]	0	0.710	0.372	0.899
**Min-Max Range**	1.214	1.046	0.9679	0.8349
	**Radius of Gyration: 10-100 ns for Protein-Ligand complex**			
**Mean ± SD**	21.8 ± 0.11	21.8 ± 0.14	22.0 ± 0.18	21.9 ± 0.13
**Min-Max Range**	0.7176	0.7979	0.9738	0.7317
	**Radius of Gyration: 20-100 ns for Protein-Ligand complex**			
**Mean ± SD**	21.8 ± 0.11	21.8 ± 0.15	22.0 ± 0.19	21.9 ± 0.13
**Min-Max Range**	0.7176	0.7979	0.9738	0.6483
	**RMSD: 10-100 ns for Ligand**			
		ATP	MMV1782355	MMV1593539
**Mean ± SD**		2.436 ± 0.24	0.8665 ± 0.37	1.487 ± 0.20
**Min-Max Range**		1.53	2.17	1.33

McsB-MMV1782355 | McsB-MMV1593539

20 ns

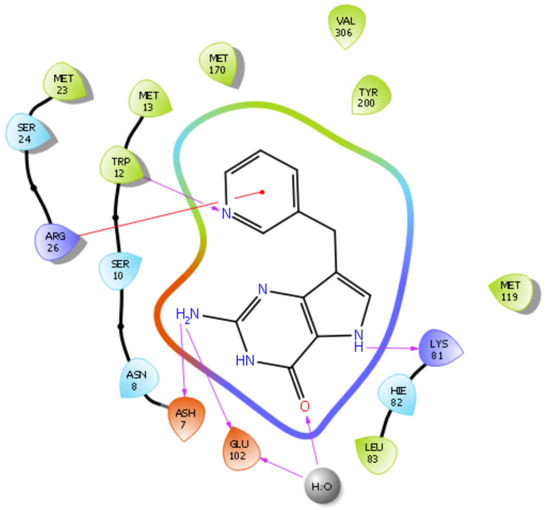

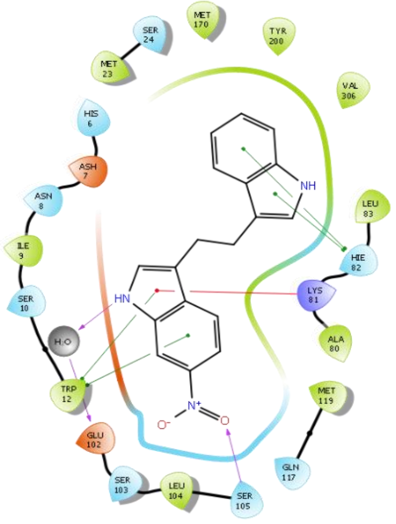

40 ns

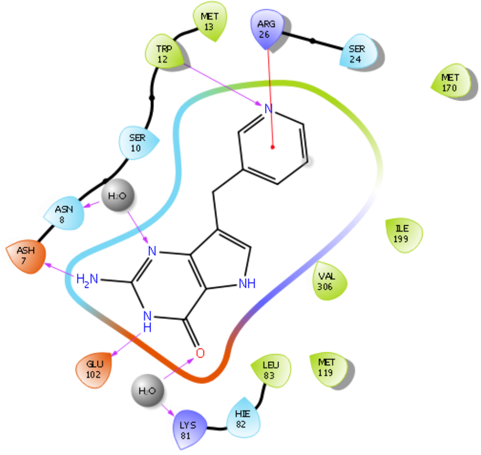

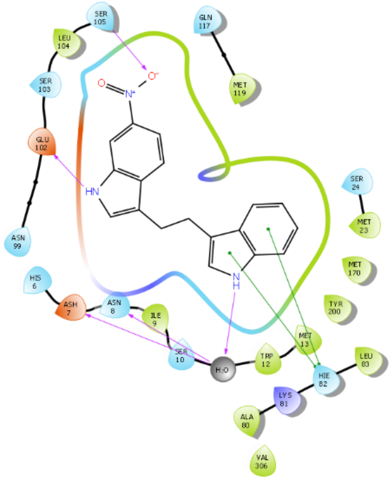

60 ns

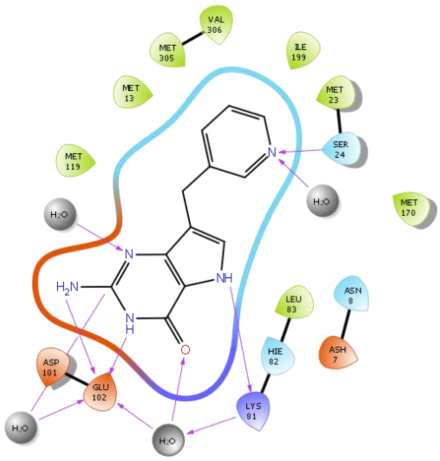

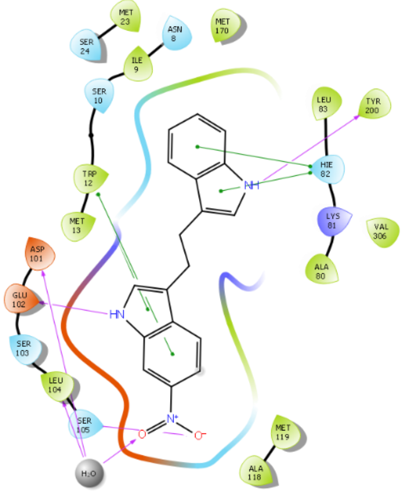

80 ns

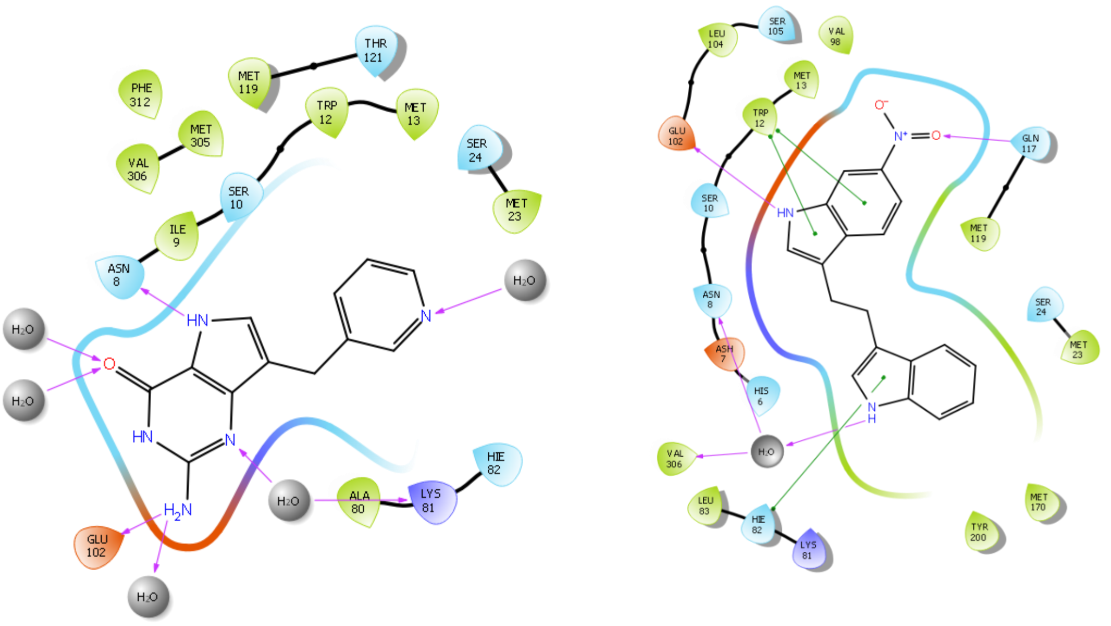

100 ns

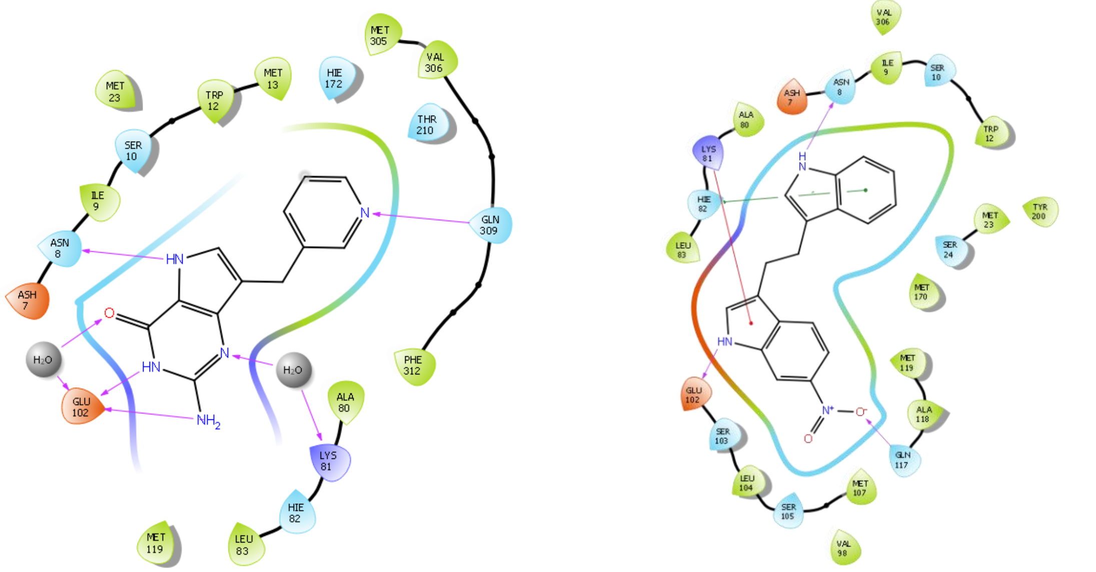

Charged (negative)	Polar	Distance	Pi-cation
Charged (positive)	Unspecified residue	H-bond	Salt bridge
Glycine	Water	Halogen bond	Solvent exposure
Hydrophobic	Hydration site	Metal coordination	
Metal	Hydration site (displaced)	Pi-Pi stacking	

Supplementary Figure S2: Ligand interaction diagrams of McsB-MMV1782355 and McsB-MMV1593539 complexes at 20, 40, 60, 80 and 100 ns after molecular dynamic simulation using Desmond.
